# Genome assembly of a potential probiotic *Bifidobacterium bifidum* BB11 strain isolated from Holstein calves

**DOI:** 10.1128/mra.00824-24

**Published:** 2024-11-13

**Authors:** Sydwell Langa, Tshifhiwa Paris Mamphogoro

**Affiliations:** 1 Gastro-Intestinal Microbiology and Biotechnology Unit, Agricultural Research Council-Animal Production, Irene, Pretoria, South Africa; University of Maryland School of Medicine, Baltimore, Maryland, USA

**Keywords:** *Bifidobacterium bifidum*, Holstein calf, bile salt tolerance, genome assembly, rumen fluid-associated bacteria, probiotic bacterium

## Abstract

Here we report the genome sequence of *Bifidobacterium bifidum* BB11 strain, isolated from the Holstein bull calf’s rumen samples. The strain tested positive for probiotic characteristics such as bile salt tolerance, adhesion, and acid tolerance. The genome size was 2,200,000 bp with the guanine and cytosine (G+C) content of 62.5%.

## ANNOUNCEMENT

Bifidobacterium was first isolated from breast-fed infant feces but has so far been discovered from various ecological niches including sewage (1) and fermented milk (2). Although *Bifidobacteria* were often isolated from calf feces (3), limited information exists on their occurrence and quantity in calf gut. Strains of *Bifidobacteria* have previously been reported to possess probiotic activities such as adhesion, bile salt tolerance (4), and acid tolerance (5).

Strain BB11 was isolated from rumen samples collected from slaughtered Holstein bull calves at the Agricultural Research Council, Irene, South Africa (25.8990023 S 28.2152523 E). The experimental procedures were performed under protocols approved by the Agricultural Research Council-Animal Production Institute Ethics Committee (APIEC24/04). A four-layered cheesecloth was used to filter the rumen fluid, which was then stored in sterile Schott bottles. The rumen fluid (1 mL) was then added to 9 mL of saline solution (0.9% wt/vol NaCl), from which serial dilutions, 10^−1^ to 10^−6^ serial dilution factors, were prepared. Subsequently, the serial dilutions were plated on De Man, Rogosa, and Sharpe (MRS) agar supplemented with the reducing agent, 0.05 g/L cysteine-HCl, and incubated anaerobically at 37°C for 48 h. Selected colonies were picked and streaked on the MRS-cysteine agar. Distinct colonies were then subcultured into MRS-cysteine-HCL broth and incubated at 37°C for 48 h. The BB11 strain tested positive for probiotic characteristics such as bile salt tolerance, adhesion, and acid tolerance (6). For the isolation and extraction of genomic DNA from 48-h-grown cultures, a Quick-DNA fungal/bacterial miniprep kit (Zymo Research, Irvine, CA) was used according to the manufacturer’s instructions. Thereafter, sequencing was performed using the Illumina NextSeq platform at Inqaba Biotechnical Industries (Pty) Ltd. (Pretoria, South Africa). The quality of the DNA was checked using the NanoPhotometer NP80 (Implen, Munich, Germany). The NEBNext Ultra II FS DNA library prep kit (New England Biolabs, Ipswich, MA) was used for DNA library preparation. The libraries were then sequenced on the Illumina NextSeq 550 platform, and a total of 3,747,545 reads generated from each sample (2 × 150-bp paired-end reads) were visualized using the KBase platform (7).

The quality of the reads was evaluated using FastQC v0.11.5 (8), and low-quality reads and sequence adaptors were removed using Trimmomatic v0.36 (9). Genome assembly was performed using SPAdes v3.13.0 (10) yielding 17 contigs with a coverage of 511×. The assembly yielded a genome sequence of 2,200,000 bp (2.2 Mb) long, with a G+C content of 62.5% and an N50 value of 310,856. Genome completeness and contamination were performed using CheckM analysis (v1.2.2) (11) and were 97.76% and 0.52%, respectively. Gene annotation was performed on RASTtk toolkit v1.073 and also following the NCBI Prokaryotic Genome Annotation Pipeline (PGAP 6.6) (12), which identified 1,748 protein-coding genes, 63 RNA genes, and 2 rRNA genes. The genome was assigned to *Bifidobacterium bifidum* via GTDB-Tk v2.3.2 (13) and had the pairwise ANI value of 98.85%. In each step, default parameters were used except where otherwise noted.

The data obtained support for future research regarding *Bifidobacterium bifidum*, phylogenetics, epidemiologies, and also mining genes coding for probiotic characteristics such as bile salt tolerance, acid tolerance, and antimicrobial resistance genes.

## Data Availability

This whole-genome shotgun project has been deposited in GenBank accession number: JAWZTF000000000, SRA accession number: SRR26857668, BioProject accession number: PRJNA1041299, and BioSample accession number: SAMN38280574.
